# Causal relations of health indices inferred statistically using the DirectLiNGAM algorithm from big data of Osaka prefecture health checkups

**DOI:** 10.1371/journal.pone.0243229

**Published:** 2020-12-23

**Authors:** Jun’ichi Kotoku, Asuka Oyama, Kanako Kitazumi, Hiroshi Toki, Akihiro Haga, Ryohei Yamamoto, Maki Shinzawa, Miyae Yamakawa, Sakiko Fukui, Keiichi Yamamoto, Toshiki Moriyama

**Affiliations:** 1 Graduate School of Medical Care and Technology, Teikyo University, Tokyo, Japan; 2 Health Care Division, Health and Counseling Center, Osaka University, Osaka, Japan; 3 Research Center for Nuclear Physics, Osaka University, Osaka, Japan; 4 Graduate School of Biomedical Sciences, Tokushima University, Tokushima, Japan; 5 Department of Nephrology, Graduate School of Medicine, Osaka University, Osaka, Japan; 6 Division of Health Sciences, Graduate School of Medicine, Osaka University, Osaka, Japan; 7 Department of Medical Informatics, Wakayama Medical University Hospital, Wakayama, Japan; University of Missouri, UNITED STATES

## Abstract

Causal relations among many statistical variables have been assessed using a Linear non-Gaussian Acyclic Model (LiNGAM). Using access to large amounts of health checkup data from Osaka prefecture obtained during the six fiscal years of years 2012–2017, we applied the DirectLiNGAM algorithm as a trial to extract causal relations among health indices for age groups and genders. Results show that LiNGAM yields interesting and reasonable results, suggesting causal relations and correlation among the statistical indices used for these analyses.

## Introduction

Metabolic syndrome (MetS), a cluster of metabolic abnormalities including visceral fat deposits, high blood pressure, elevated fasting blood glucose, and atherogenic dyslipidemia, presents a major public health challenge worldwide [1]. Although the precise mechanisms underlying MetS remain unclear, multiple reports have described that a complex interaction among genetic, metabolic, and environmental factors contributes to its pathogenesis [2]. Different populations have widely varied prevalence of MetS with different severities of various components [3]. To establish an effective strategy for preventing MetS in certain populations, its complex interactions must be clarified. After clarifying those interactions, strategic priorities can be assigned. Because of the complexity of MetS, few methods have been used to identify and prioritize its contributing factors.

Using access to large amounts of health checkup data obtained in Osaka prefecture during fiscal years 2012–2017, we are striving to ascertain the causes of diseases and to prevent severe illness. Checkup data include many health indices, all of which are expected to be interconnected through complicated relations. As one might expect, ascertaining which health indices influence other indices is important and necessary. Eventually such knowledge can be related to the prevention and treatment of severe diseases. Statistics indicating the quantities of MetS cases and the distributions of people who have not yet reached the stage of MetS are typically available. Nevertheless, assessing big data of health checkups presents great difficulties for the extraction of causal relations, for indication of which health indices are contributors to other indices, and for indication of which indices are more independent of others.

Health indices are usually mutually related, as suggested by correlations among various indices. Longitudinal studies such as cohort studies using randomized controlled trials (RCTs) and propensity scores have been conducted to infer causal relations of these variables [4]. However, cross-sectional research has attracted attention recently for causal inference such as Mendelian Randomization, but it is impossible without single nucleotide polymorphism (SNP) information [5, 6].

Recently, a powerful mathematical algorithm was introduced to infer causal relations among variables based solely on statistical data. This Linear non-Gaussian Acyclic Model (LiNGAM) was introduced by Shimizu and his collaborators [7–9]. In fact, the LiNGAM algorithm is a powerful method for extracting causal relations among variables solely from statistical data using probability distributions of variables that are, in general, non-Gaussian. To use LiNGAM, one must use large amounts of data for reliable extraction of causal relations among many variables. Moreover, a powerful computer with large capacity for computer storage must be used to attain adequate rates of execution of the numerical calculations.

In typical situations, big data analyses have been conducted using multiple regression models or some machine learning models such as support vector machine and random forest. Nevertheless, these correlation analyses do not clarify the causality of variables. Widely applied models used to infer causality are structural equation models [10] and Bayesian networks [11]. A particular case of those models, LiNGAM, presents the benefit of being useful to build causal diagrams without prior knowledge. One type of LiNGAM algorithm, DirectLiNGAM, uses regression to infer causal ordering from multivariate data [8, 12].

As the first trial, we present results of DirectLiNGAM analysis of health checkup data from Osaka prefecture. The purpose of this paper is to describe DirectLiNGAM and to elucidate this method’s suitability for health checkup data analyses. Section 2 presents a brief description of the DirectLiNGAM algorithm in the context of dealing with big data. Section 3 introduces health checkup data for DirectLiNGAM analysis. In Section 4, we present numerical results obtained using the Osaka health checkup data. Section 5 explains a comparison of these results with those obtained using other algorithms. Section 6 presents a summary of the results and presents some discussion in support of future studies using the DirectLiNGAM algorithm.

## Method

Herein, the DirectLiNGAM algorithm is briefly described conceptually using details presented in the literature [7–9, 12]. We first describe how a causal relation between two variables is obtained. For cases involving many variables, one must know how to obtain the first variable among all the other variables using the causal relation between two variables. Subsequently, this method is repeated for all causal orders of all variables. We specifically address statistical distributions with errors. Therefore, we introduce a bootstrap algorithm for robustness of the causal relations and their correlations.

### Causal relation of two variables

First, we discuss the causal relation of two variables expressed as *x*_1_ and *x*_2_. We express a probability distribution of two variables as *p*(*x*_1_, *x*_2_). For statistical data, the probability distribution corresponds to the density of points in a scatter plot with *x*_1_ and *x*_2_ axes. For *x*_1_ as the source of *x*_2_, the causal relation of two variables demands the following relations:

$$\begin{array}{c} x_{1}=e_{1}\;,x_{2}=b_{21}x_{1}+e_{2}\;. \end{array}$$ (1)

Distribution *e*_1_ and the residual distribution *e*_2_ = *x*_2_ − *b*_21_ *x*_1_ are independent, which means that the probability distributions of *e*_1_ and *e*_2_ are separable as

$$\begin{array}{c} p\left(e_{1},e_{2}\right)=p\left(e_{1}\right)p\left(e_{2}\right)\;, \end{array}$$ (2)

where *p*(*e*_1_) and *p*(*e*_2_) respectively represent distribution functions for *e*_1_ = *x*_1_ and *e*_2_ = *x*_2_ − *b*_21_ *x*_1_.

In reality, we deal with statistical data. Any distribution includes statistical fluctuation. Any statement of independence includes some ambiguity. All variables have different dimensions and different distributions with some average value and standard deviation. For comparison of any pair of distributions, we first standardize all distributions with zero average value with a standard deviation one. Hereinafter, all distributions are standardized unless noted otherwise. Given this preparation, the Kullback–Leibler (KL) divergence *D*(*p*||*q*) for two probability distributions, *p* and *q* [13], can then be used for two variables *x*_1_ and *x*_2_ of the example given above to find ordering of the two variables. We compare two divergences as

$$\begin{array}{c} D_{1}(p\left(x_{1},x_{2}-b_{21}x_{1}\right)||p\left(x_{1}\right)p\left(x_{2}-b_{21}x_{1}\right))\; \end{array}$$ (3)

and

$$\begin{array}{c} D_{2}(p\left(x_{2},x_{1}-b_{12}x_{2}\right)||p\left(x_{2}\right)p\left(x_{1}-b_{12}x_{2}\right))\;. \end{array}$$ (4)

Using LiNGAM, one can compare the two divergences as *m*_12_ = *D*_1_ − *D*_2_. If *m*_12_ is negative, then *D*_1_ is smaller than *D*_2_. It can be said that *x*_1_ is more likely to be the source of *x*_2_ than the other way around. Ostensibly, *x*_2_ is more likely to be the source of *x*_1_ if *m*_12_ is positive. If this *m*_12_ is approximately equal to zero, then the causal relation of the two variables is fragile: in such a case, causality between the two variables cannot be inferred.

Divergence *D* must be calculated in the actual data case. With the DirectLiNGAM algorithm, we use the following quantity designated as two-variable entropy.

$$\begin{array}{c} H\left(x_{1},x_{2}\right)=-\;\int \int \;p\left(x_{1},x_{2}\right)\;\text{log}\;p\left(x_{1},x_{2}\right)dx_{1}dx_{2}\; \end{array}$$ (5)

We also use one-variable entropy as

$$\begin{array}{c} H\left(x_{i}\right)=-\int p\left(x_{1},x_{2}\right)\;\text{log}\;p\left(x_{i}\right)dx_{1}dx_{2}=-\int p\left(x_{i}\right)\;\text{log}\;p\left(x_{i}\right)dx_{i}\;, \end{array}$$ (6)

for *i* = 1, 2. We can then express divergence *D* using the entropies defined above as

$$\begin{array}{c} D\left(x_{1},x_{2}\right)=-H\left(x_{1},x_{2}\right)+\sum_{i=1}^{2}H\left(x_{i}\right)\;. \end{array}$$ (7)

Using this definition of the divergence in terms of entropy, one can write the relation *m*_*ij*_ as

$$\begin{array}{c} m_{ji}=\left[H\left(x_{j}\right)+H\left(r_{i}^{\left(j\right)}\right)\right]-\left[H\left(x_{i}\right)+H\left(r_{j}^{\left(i\right)}\right)\right]\;, \end{array}$$ (8)

where $r_{i}^{\left(j\right)}$ represents the residual variable $r_{i}^{\left(j\right)}=x_{i}-b_{ij}x_{j}$. The two reciprocal entropy terms $H(x_{j},r_{i}^{\left(j\right)})$ and $H(x_{i},r_{j}^{\left(i\right)})$ can be verified to cancel each other in *m*_*ji*_. An approximation for the entropy can be introduced to speed up all the LiNGAM calculations as

$$\begin{array}{c} H\left(x\right)=\frac{1}{2}\left(1+\text{log}2\pi \right)-79.047{\left(E\left[\text{log}\;\text{cosh}\;x\right]-0.37457\right)}^{2}-7.4129{\left(E\left[x\;\text{exp}\;\left(-x^{2}/2\right)\right]\right)}^{2}\;, \end{array}$$ (9)

where *E*(*y*) denotes the average of *y* distribution. All numerical values are obtained numerically, as described in an earlier report [14]. These terms reflect the amount of non-Gaussian property of the *x* distribution. Therefore, in LiNGAM, one uses the non-Gaussian property of all the probability distributions.

### Ordering of many variables

By calculating *m*_*ij*_, we can order two variables. To obtain the first variable among all *p* variables, one can repeat all the comparisons calculating *m*_*ij*_. With DirectLiNGAM, one can use the following *M* criterion to select the first variable among them.

$$\begin{array}{c} M\left(x_{i};U\right)=-\sum_{j\in U}\text{min}{\left(0,m_{ji}\right)}^{2}\; \end{array}$$ (10)

In that equation, *U* represents a group of all the suffixes as *U* = {1, 2.., *p*}. In addition, *M* is zero if *m*_*ji*_ are positive for all variables *j* for a variable *i*. This is the ideal case because variable *i* is the source of all the other variables. However, in some cases, *m*_*ji*_ appears to be negative. Consequently, *M* becomes negative and finite. In this case, this criterion demands that *M* be closest to zero. Comparing *M*(*x*_*i*_) for all variables *i*, the first variable can be chosen among all the variables by finding *i* with the maximum *M* value. This variable is redesignated as *x*_1_; all the rest are redesignated as *x*_2_.., *x*_*p*_.

The next step requires that the effect of the first variable be removed from those of all the other variables as

$$\begin{array}{c} x_{i}^{\prime }=x_{i}-\frac{cov(x_{i},x_{1})}{var(x_{1})}x_{1}\;, \end{array}$$ (11)

for *i* = 2.., *p*. We standardize new variables $x_{i}^{\prime }$ and repeat the procedure described above to ascertain the first variable $x_{i}^{\prime }$ among all remaining variables in *U* = {2.., *p*} by comparing the *M* values. This procedure is then repeated numerous times to ascertain the causal order of all variables. The order of the original *i* variable can then be found as *k*(*i*), where the *i* variable is ordered at the *k*-th variable.

One can then find the structure causal matrix *B* using order *k*(*i*). A multiple regression method is applied as

$$\begin{array}{c} x_{i}=\sum_{j\in A_{i}}b_{ij}x_{j}+e_{i}\;, \end{array}$$ (12)

where

$$\begin{array}{c} A_{i}=\left\{j|k\left(j\right)<k\left(i\right)\right\}\;. \end{array}$$ (13)

In principle, all *B* matrix elements can be calculated.

When used along with many variables, however, this method presents some instability in calculations. Therefore, constraint terms are introduced so that multiple regression calculations become stable using the Lagrange method. To avoid unnecessary confusion of notation, we consider a linear multiple regression of variable *y* with numerous data points *N*, written as *y*_*k*_ with *k* = 1.., *N*. We have multiple variables *x*_*i*_ for *i* = 1.., *p* to make regression of *y*, where each variable *i* has *N* data points, *x*_*ki*_ with *k* = 1.., *N*. The expression above corresponds to minimization of the following function with respect to the weight coefficients *w*_*i*_ with *i* = 1.., *p*.

$$\begin{array}{c} l=\frac{1}{N}\sum_{k=1}^{N}{\left(y_{k}-\sum_{i=1}^{p}x_{ki}w_{i}\right)}^{2} \end{array}$$ (14)

In this case, an instability problem, a so-called norm problem, arises when some variables have similar distributions. A standard method to avoid the instability problem is to regularize the function to be minimized. We adopt the elasticnet method instead of the AdaptiveLasso method used by Shimizu et al. [8]. Using the elasticnet method, the following function is minimized.

$$\begin{array}{c} l^{\prime }=l+\lambda [\sum_{i=1}^{p}\left(\alpha \right|w_{i}\left|+\left(1-\alpha \right)\frac{w_{i}^{2}}{2}\right)] \end{array}$$ (15)

By choosing constraint parameters λ and *α* for the grid search, a stable solution for *w*_*i*_ can be found.

After returning to the original notation, one can repeat the multiple regression analysis for all ordered variables to obtain structure causal (SC) matrix *B* with matrix elements that are finite only in the lower triangle of the *B* matrix.

$$\begin{array}{c} \left(\begin{array}{c} x_{1}\\ x_{2}\\ x_{3}\\ ..\\ x_{p} \end{array})=(\begin{array}{ccccc} 0 & 0 & .. & 0 & 0\\ b_{21} & 0 & .. & 0 & 0\\ b_{31} & b_{32} & .. & 0 & 0\\ .. & .. & .. & .. & ..\\ b_{p1} & b_{p2} & .. & b_{p(p-1)} & 0 \end{array})(\begin{array}{c} x_{1}\\ x_{2}\\ x_{3}\\ ..\\ x_{p} \end{array})+(\begin{array}{c} e_{1}\\ e_{2}\\ e_{3}\\ ..\\ e_{p} \end{array}\right) \end{array}$$ (16)

One can then infer a causal relation with ordering of the variables and the structure causal matrix *B*, the matrix elements of which provide information for how large causal variables influence the resulting variables.

### Bootstrap algorithm of statistical robustness

The DirectLiNGAM algorithm is written to elicit causal relations among variables for a large dataset. Nevertheless, all datasets can be expected to include some statistical error. We must estimate how robust the causal relations are among the variables. The standard method is the bootstrap algorithm explained below.

Presuming that big data exist with numerous samples for several variables, where the sample number is *N*, we choose *N* samples randomly one-by-one using random sampling with replacement, where we return a chosen sample in one round for the next round and continue this process *N* times. The samples then constitute one dataset. This restore–extraction process is then repeated *n* times to yield *n* datasets. Subsequently, the DirectLiNGAM algorithm is applied for each dataset to obtain a probability of causal relations. This is a random process. Therefore, *n* datasets differ. In a dataset, several samples are used in a multiple fashion. Several other samples are not used at all. Using the so-created *n* datasets yields information about the degree of robustness of the ordering of variables and about errors of correlation among variables.

## Health checkup data of Osaka prefecture

The LiNGAM algorithm was applied to National Health Insurance (NHI) and Senior Elderly Insurance (SEI) health checkup data in Osaka prefecture. For this study, we were provided several datasets including health checkup data, medical receipt data, care receipt data, and their related details for the six fiscal years of 2012–2017.

The Ethics Committee of Health and Counseling Center, Osaka University (IRB Approval Number 2018-9) and Osaka University Hospital (IRB Approval Number 19073) approved the study protocol. All procedures used for studies involving human participants were conducted in accordance with the 1964 Declaration of Helsinki and its later amendments or comparable ethical standards. Informed consent was not obtained from participants because all data were anonymized, according to Japanese Ethical Guidelines for Medical and Health Research Involving Human Subjects enacted by the Ministry of Health, Labour and Welfare of Japan (https://www.mhlw.go.jp/le/06-Seisakujouhou-10600000-Daijinkanboukouseikagakuka/0000080278.pdf; https://www.mhlw.go.jp/le/06-Seisakujouhou-10600000-Daijinkanboukouseikagakuka/0000153339.pdf). Although all data were anonymized, we are strictly prohibited by owners of these data from opening the entirety of the data to the public.

### Details of health checkup data

For these analyses conducted for the first reported trial of the DirectLiNGAM algorithm, health checkup data of fiscal year 2016 were used. The health checkup data include information for 679 351 IDs. For our analyses, 11 items were selected: systolic blood pressure (sBP), low-density lipoprotein cholesterol (LDL), high-density lipoprotein cholesterol (HDL), triglyceride (TG), glutamic oxaloacetic transaminase (GOT), gamma-glutamyl transpeptidase (*γ*GT), glutamic pyruvic transaminase (GPT), body mass index (BMI), fasting blood glucose level (fBG), hemoglobin A1c (HbA1c), and height. After removing IDs without values (NA) for all 11 items, we assumed some numbers as NA if numbers in each item had been introduced by mistake. Finally, outliers were removed: they were IDs for which numbers were very large or very small, representing 0.05% of all the data on each side. The resultant number of IDs was 588 060. Percentiles for all 11 items are shown in Table 1.

**Table 1:** Percentile values of respective indexes, with minimum, maximum, mean values, and standard deviations.

Index	Min	25%	50%	75%	Max	Mean	Std
BMI, kg/m^2^	13.8	20.5	22.6	24.8	40.9	22.82	3.35
GOT, IU/L	11	19	22	27	177	24.33	9.32
GPT, IU/L	5	13	17	23	186	20.29	12.18
HDL, mg/dL	25	52	62	74	144	63.74	16.65
HbA1c, %	4.5	5.4	5.6	5.9	13.1	5.72	0.63
LDL, mg/dL	32	102	121	142	260	122.67	30.51
TG, mg/dL	26	70	95	131	1009	110.45	66.37
fBG, mg/dL	62	88	94	103	310	98.53	19.28
height, cm	129.1	151	157.3	164.5	186.1	157.87	9.17
sBP, mmHg	81	118	130	140	207	129.64	17.49
*γ*GT, IU/L	8	16	22	36	804	33.97	40.70

The numbers of samples (IDs) for each age group and gender are presented in Table 2. The numbers of samples were greater than 30,000 for both genders for people in their 60s, 70s, and 80s. We present the results of those cases with more than 30,000 samples. Additionally, we discuss results obtained for smaller samples as in those in their 50s for comparison with those obtained from larger samples.

**Table 2:** Numbers of IDs by age group and gender.

Age	Men	Women
30–39	374	429
40–49	22 333	23 539
50–59	20 316	26 654
60–69	69 892	109 529
70–79	97 327	131 036
80–89	32 594	46 906
90–99	2147	4881
100–109	17	86

### Estimation of causal order

The causal orders for all age groups and genders are calculated because the health indices of men and women differ greatly. The health indices are influenced also by age. We are interested in observing causal relations among health indices in each age group. Depending on the sample number, we obtain statistically desirable and non-desirable cases. To demonstrate the LiNGAM analysis procedures and the results, the case of women in their 70s is explained first: its sample number is 131,036: The largest among all cases.

**Fig 1:**
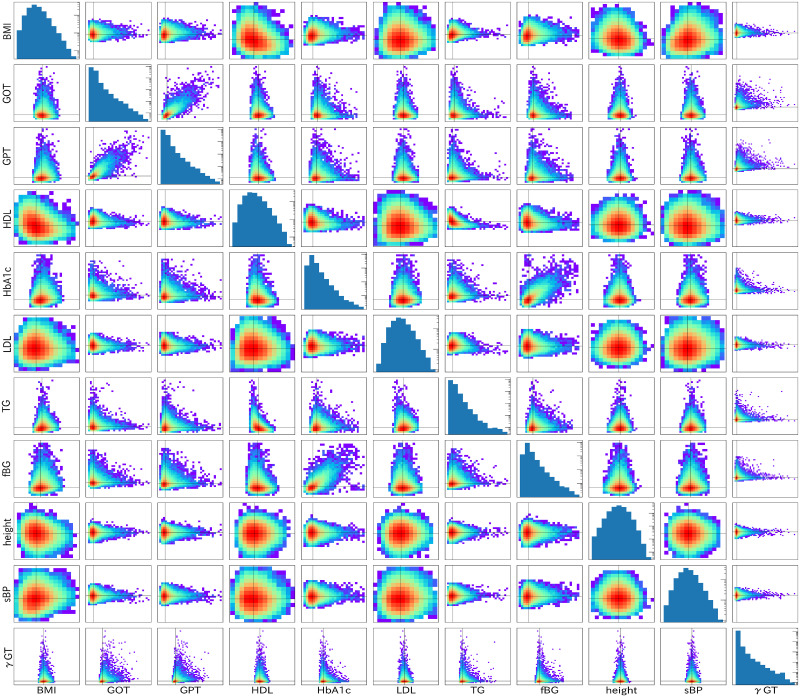
Basic correlations among health indices and distributions for individual indices are shown for women in their 70s. Basic correlations among health indices are presented on a log-scale density plot in non-diagonal slots. Distributions of health indices are presented in the log-scale histogram in the diagonal slots.

First, we present basic correlation among health indices and the distributions of health indices for women in their 70s. We present basic correlation among health indices on a log-scale density plot in Fig 1. A health index for each correlation figure is shown on the vertical axis as a function of a health index shown on the horizontal axis. In the diagonal slots, we present the distribution of the health index in each figure, where the vertical axis represents the frequency and the horizontal axis represents the corresponding index. Also, the number of people in each category is shown on the vertical axis by a histogram. This figure presents all details of the present health checkup data. Further LiNGAM analyses use only these correlation distributions.

Several interesting features are apparent in this figure. One is strong correlation among the health indices. They are HbA1c-fBG pair for glucose in the blood and GOT-GPT pair for liver indices. The correlation slope is almost 45 degrees for the standardized indices, which indicates that these two paired indices convey almost identical information. The others are almost round correlations for several correlation plots for LDL, height, and sBP. These round correlations reflect that these paired indices are almost mutually independent.

**Fig 2:**
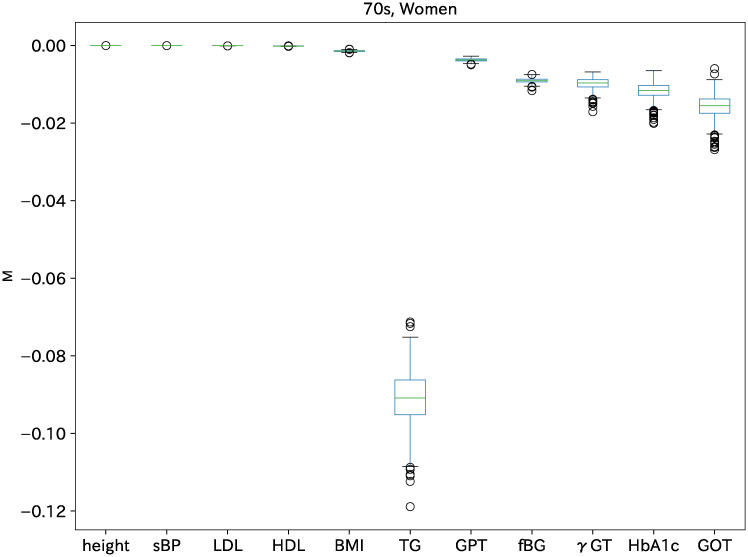
*M* distribution of various indices for women in their 70s.

The *M* distribution of 1000 trials for all indices (variables) is shown first to ascertain the first index among all indices in Fig 2. For this calculation, the Lagrange constraint parameters λ and *α* are fixed optimally so that the signals of the orderings are apparently the best. The largest *M* among all the indices is expected to be the first variable. Indices close to *M* = 0 are height, sBP, LDL, HDL, and BMI, which are expected to come earlier in the causal hierarchy. Those indices with smaller *M* are TG, fBG, HbA1c, *γ*GT, GPT, and GOT, which are expected to come later in the causal hierarchy. We repeat 1000 trials and fix the causal order. The frequencies of various causal orders are shown in Table 3. The most frequent order shown in the top row appears 958 times among 1000 trials. The next order appears only 16 times among all 1000 trials, as shown in the second row of the same table.

**Table 3:** Frequencies of orders of various indices in 1000 trials for women in their 70s.

Count	1	2	3	4	5	6	7	8	9	10	11
958	height	sBP	LDL	HDL	BMI	TG	GPT	fBG	*γ*GT	HbA1c	GOT
16	height	sBP	LDL	HDL	BMI	TG	fBG	GPT	*γ*GT	HbA1c	GOT
10	height	sBP	LDL	HDL	BMI	TG	fBG	*γ*GT	HbA1c	GPT	GOT
6	height	sBP	LDL	HDL	BMI	TG	fBG	HbA1c	GPT	*γ*GT	GOT

The four frequent orders of indices appearing in our analysis are listed from the top to the bottom rows. The indices are arranged from the earliest to the latest in each causal order from left to right columns.

The most frequent order is height, sBP, LDL, HDL, BMI, TG, GPT, fBG, *γ*GT, HbA1c, and GOT. The order of fBG and GPT is replaced in the second row because they are fundamentally independent, as portrayed in the correlation plot in Fig 1. Roughly speaking, the members of the group of glucose indices (fBG and HbA1c) are replaced by the group of liver indices (GPT, *γ*GT, and GOT) when comparing the most frequent order with the third and fourth orders. It is noteworthy that the order of indices in the glucose index group is unchanged; the order in the liver group differs between *γ*GT and GPT. This replacement is reasonable because correlation of the glucose index group and the liver index group is exceedingly weak. It is noteworthy that the height comes in the early stage in the causal order. The most frequent causal order is expected to be very robust when the number of samples is large. Statistically desirable results are not obtained if one performs the same analysis for age groups with fewer samples *N*. Apparently, more than 30 000 samples are necessary to obtain satisfactory results from the present LiNGAM analysis.

### Estimation of partial regression coefficients

The partial regression coefficients in the structure of causal *B* matrix can be estimated next. For this presentation, one should know the causal order already. For women in their 70s, a sufficient number of samples is available. For the most frequent order, 958 cases exist. Distributions of partial regression coefficients can be provided for the ordered indices. We present the B matrix in Table 4, where the matrix elements (upper numbers) and their standard deviations (lower numbers with ± in front) are shown, with probability distributions which approximate the Gaussian distributions. The standard deviations are 0.002–0.005. The indices are standardized. Therefore, the regression coefficient of 0.1 indicates that the target index changes by 0.1 of the standard deviation of the target index as the source index changes by 1 standard deviation of the source index. For observation of the causal order and the correlations among all indices, we indicate those relations by arrows with thickness depending on their correlation in the following causal figure for women in their 70s.

**Table 4:** Correlation coefficients with standard deviation in the B matrix.

Index	height	sBP	LDL	HDL	BMI	TG	GPT	fBG	*γ*GT	HbA1c	GOT
height											
sBP	−0.030 ±0.003										
LDL	0.025 ±0.003	0.045 ±0.003									
HDL	−0.008 ±0.003	−0.030 ±0.003	−0.019 ±0.003								
BMI	−0.126 ±0.003	0.151 ±0.003	−0.015 ±0.003	−0.291 ±0.002							
TG	0.020 ±0.002	0.054 ±0.003	0.085 ±0.003	−0.421 ±0.002	0.107 ±0.003						
GPT	0.027 ±0.003	0.006 ±0.003	−0.060 ±0.003	0.020 ±0.003	0.175 ±0.004	0.098 ±0.004					
fBG	0.028 ±0.003	0.066 ±0.003	−0.038 ±0.003	−0.031 ±0.003	0.140 ±0.003	0.089 ±0.004	0.109 ±0.004				
*γ*GT	−0.002 ±0.002	0.008 ±0.003	−0.018 ±0.003	0.061 ±0.004	0.016 ±0.003	0.107 ±0.005	0.381 ±0.005	0.046 ±0.004			
HbA1c	−0.014 ±0.002	−0.028 ±0.002	−0.002 ±0.002	−0.044 ±0.002	0.033 ±0.002	0.012 ±0.003	0.041 ±0.003	0.687 ±0.004	−0.021 ±0.003		
GOT	−0.037 ±0.002	0.009 ±0.002	−0.026 ±0.002	0.022 ±0.002	−0.089 ±0.002	−0.036 ±0.002	0.801 ±0.004	−0.031 ±0.003	0.064 ±0.005	−0.043 ±0.003	

The indices are ordered by their causal order. The row indices are influenced by the column indices.

### Causal diagram

**Fig 3:**
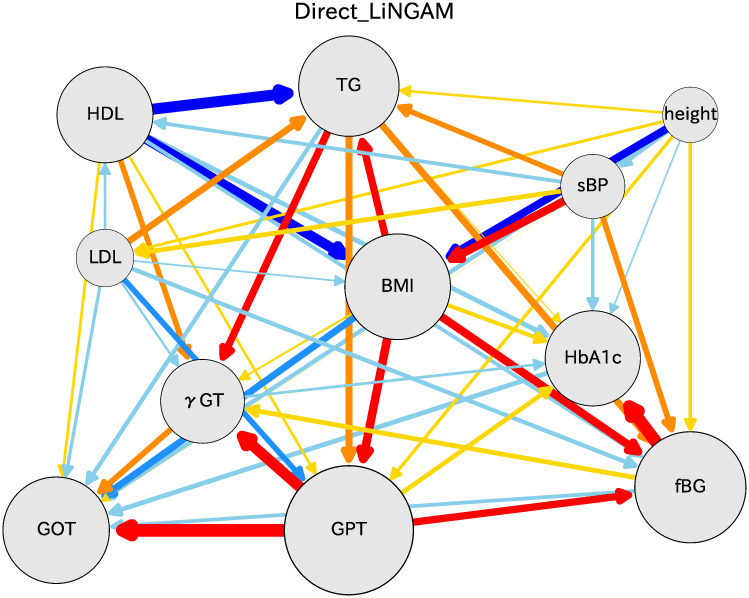
Causal diagram for women in their 70s. Arrows indicate the causal order between two connected indices. The arrow bar size is proportional to the logarithm of absolute value of the partial regression coefficient. The color depends on the partial regression coefficient *b*: red for *b* ≥ 0.1, orange for 0.1 > *b* ≥ 0.05, yellow for 0.05 > *b* > 0, sky blue for 0 > *b* ≥ -0.05, blue for -0.05 > *b* ≥ -0.1, and deep blue for *b* <-0.1.

One can obtain the causal order and the partial regression coefficients using the DirectLiNGAM algorithm. Several means exist to present the causal relation results. That shown in Fig 3 includes all located indices to clarify the causal relations among various indices. The circle radius is obtained using the sum of absolute values of regression coefficients going in and out of the index. This figure shows lines with arrows and colors with thicknesses chosen in accordance with the logarithm of absolute values of the partial regression coefficient. The arrows represent causal relations between two connected indices. Five colors represent the strength of correlation in rainbow color order. The blue color side (deep blue, blue, and sky blue) shows that a target index decreases as an independent variable increases, whereas the red color side (red, orange, yellow) shows that a target index increases as an independent variable increases. Here, we have removed lines that are not statistically significant, as inferred using the Bonferroni criterion with multiple regression analysis.

Fig 3 shows that causal relations were inferred from earlier indices in the causal hierarchy for the most frequent order. Height influences BMI with coefficient −0.126; that sign is reasonable because BMI is reciprocally proportional to the squared height. Results show that sBP influences BMI with the coefficient 0.151, which represents an important causal relation because sBP is one source of increasing BMI. Results demonstrate that LDL influences TG with coefficient 0.085 and GPT with -0.060. HDL strongly influences reduction of BMI with coefficient 0.291, and simultaneously influences TG with a large coefficient of 0.421. This finding in the present analysis is extremely important for health guidance: HDL should be emphasized to maintain the health of individuals. TG is influenced by HDL and with a small coefficient by BMI, and influences GPT with small correlation. Finally, BMI seems to hold a role as a key index among all indices. BMI influences the glucose indices as fBG and HbA1c, and influences liver indices as GPT and *γ*GT.

The association of GPT with GOT is strong: a strong relation exists between GPT on GOT. fBG is influenced by BMI and GPT, but it influences HbA1c. The association of fBG with HbA1c is also strong. These strong correlations in the glucose indices and in the liver indices are already apparent in the basic correlation plots presented in Fig 1. The results reported herein suggest that GPT and GOT are almost identical indices in terms of liver status. Regarding glucose, both fBG and HbA1c are similar indicators of the blood glucose amount. These results are extremely important for considering the source of risk indices of severe illness.

## Numerical results

**Fig 4:**
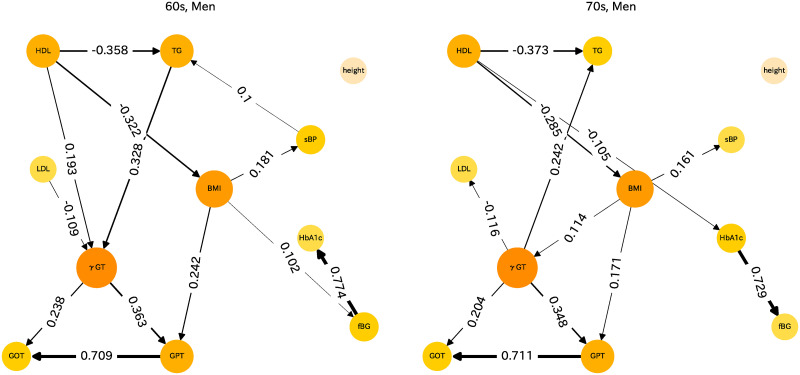
Causal diagram for men in their 60s and 70s.

**Fig 5:**
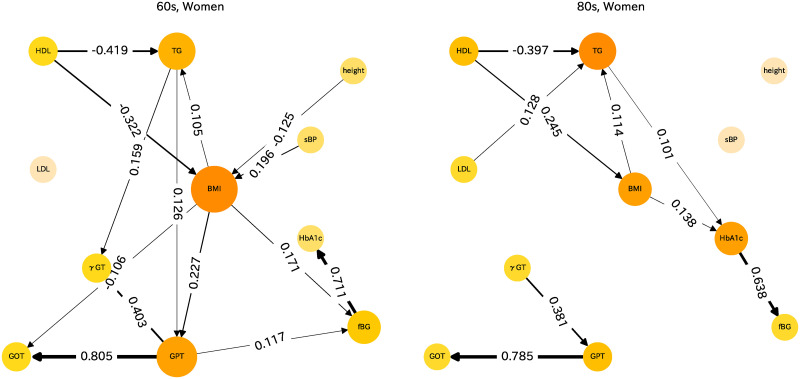
Causal diagram for women in their 60s and 80s.

For men and women of other age groups, LiNGAM analysis results can also be presented. Health indices differ greatly between those of men and women and also among age groups. Therefore, the causal relations for men and women for the respective age groups are assessed separately. Causal diagrams are presented for men in Fig 4 and for women in Fig 5. These figures show causal relations with absolute values of the partial regression coefficients of more than 0.1 merely for heuristic reasons.

### Causal diagram for men

We next address the causal relations of men in their 60s, shown as the left panel of Fig 4. The causal diagrams are mostly similar to those for women in their 70s. The fact that HDL strongly influences TG and BMI is unchanged. BMI is influenced by sBP; it influences HbA1c and GPT. Also, TG influences *γ*GP, which is apparently the gateway index of the liver indicators. For men in their 70s, shown as the right panel of Fig 4, the causal relations are quite similar to those of men in their 60s. Here, *γ*GT influences TG. For men also, HDL has a strong beneficial effect on TG and BMI.

### Causal diagram for women

Causal relations of women in their 60s are shown as the left panel of Fig 5. The causal diagrams resemble those of women in their 70s. The relation of HDL to TG and BMI is robust. BMI is influenced by sBP and influences fBG and GPT. TG influences both *γ*GT and GPT. For women in their 80s, as shown as the right panel in Fig 5, the character of the causal relations closely resembles that found for women in their 60s, but it becomes simpler. The connection between the GPT group and other indices becomes weaker for women of this age group than for women of other age groups.

### Sample number dependence of DirectLiNGAM

**Fig 6:**
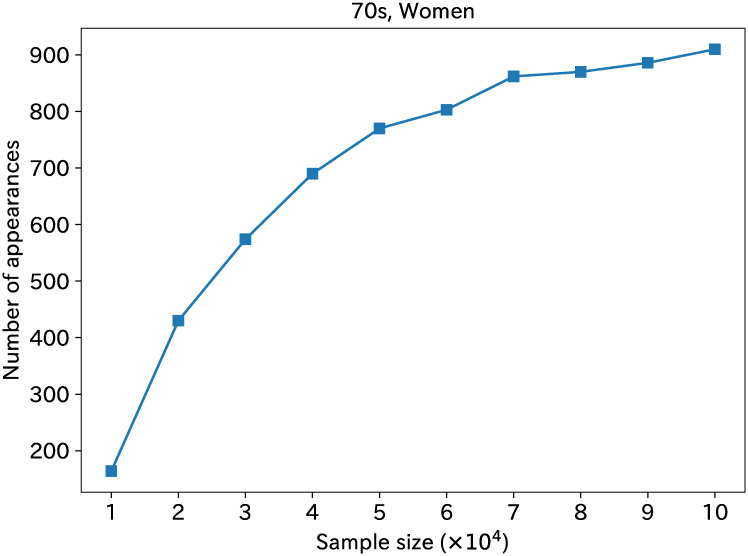
Number of cases in the top ordering, shown as a function of the sample size. The number of cases is expressed as the frequency on the vertical axis with the full amount of 1000.

We made calculations of several groups with fewer samples. This sample size reduction was performed by reducing samples randomly for women in their 70s. Fig 6 shows that the number of cases in the top ordering can be presented as a function of the sample size. The frequency in the top ordering decreases concomitantly with a decreasing number of samples. When the sample size is about 20,000, the frequency becomes about 400 out of 1000. When the frequency is lower, the most probable ordering appears fewer times. Therefore, larger errors become apparent in the causal order and correlation among statistical variables. It is noteworthy that the causal order in the top ordering is unchanged, even for the 10,000 sample size reduced from the full sample size.

### Reduction of the number of variables

**Fig 7:**
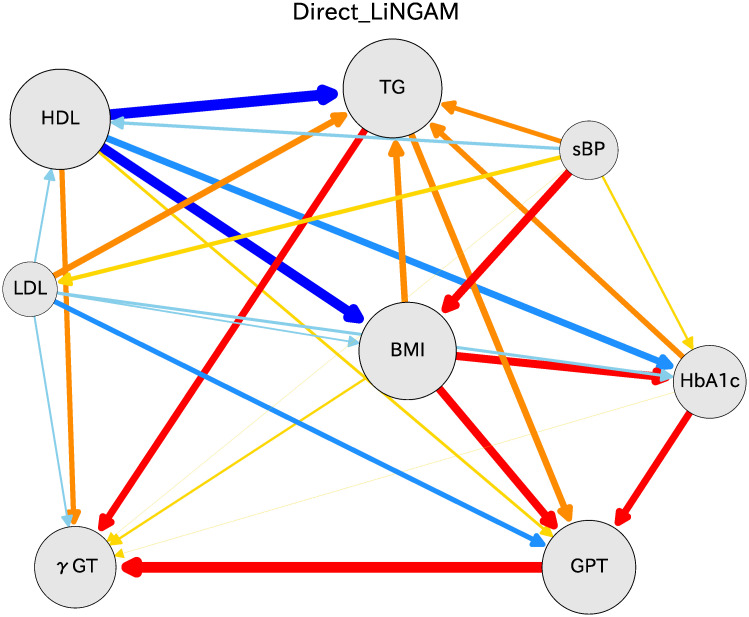
Causal diagram for women in their 70s. This figure uses 8 indices instead of the 11 indices shown in Fig 3. The causal relations and the correlation coefficients are much more robust than in the case of 11 indices. The colors and the arrow thicknesses are used for causal relations, as depicted in Fig 3.

We identified interesting causal relations among 11 variables in the Osaka prefecture health checkup data. To elucidate the effects of fewer variables, GOT, fBG, and height were dropped. For the 11 variables, the order between HbA1c and fBG was fragile. We removed fBG and thereby obtained much more stable ordering than in the case with 11 variables. GOT was found every time in last place in the causal order. Therefore, we dropped GOT. After doing so, the correlation coefficients were more stable, with much less statistical error. One result for the eight-variable case is shown: women in their 70s in Fig 7. The results are fundamentally equivalent to those obtained for the case with 11 variables.

We obtain fundamentally identical information for the 8-variable case to that obtained for the 11-variable case. Regarding the causal relations, the removal of fBG and GOT clarify these relations. In addition, HDL influences BMI and TG. If one regards BMI as a key index, then it is influenced by sBP among health indices, whereas BMI influences TG, HbA1c, and GPT and *γ*GT. Results show that sBP is quite independent of other indices. However, many other indices influence HbA1c. Among the eight health indices, the liver indices (*γ*GT and GPT) are at the bottom of the causal order.

Among the selected 11 variables in this study, neither renal functions nor urinary proteins were included. Urinary proteins, which might be unmeasured confounders, were categorical variables. They could not be analyzed using LiNGAM. In addition, creatinine was not included in this analysis because it is not a mandatory item for a specific health checkup. Further examination is required from future collection of these data.

## Comparison with ICA-LiNGAM

**Fig 8:**
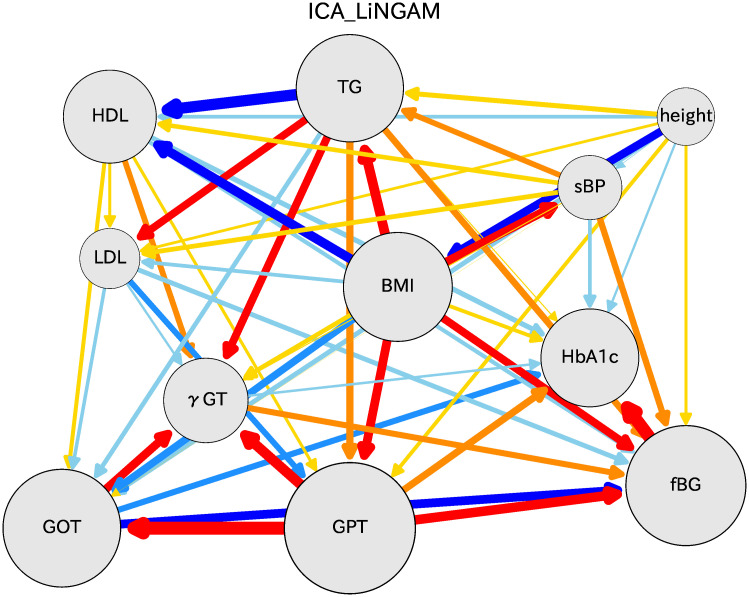
Causal diagram for women in their 70s. We use the ICA-LiNGAM algorithm for this causal relation. For this figure, we take 11 indices as DirectLiNGAM in Fig 3. The arrow colors and the thicknesses are used for the causal relations, as depicted in Fig 3.

We have been using the DirectLiNGAM algorithm to evaluate health checkup data. Several algorithms are useful to assess causal relations and partial regression coefficients. Comparing the results to those obtained using other similar algorithms is important. To this end, we chose the ICA-LiNGAM algorithm reported by Shimizu *et al*. [7]. The ICA-LiNGAM software is available from the ‘pcalg’ package for R [15, 16]. The same health checkup data as those for women in their 70s were used. After 1000 iterations of bootstrap calculations, the results were obtained as shown in Fig 8. The significance level is set using the Bonferroni method. The arrow thickness is fixed by correlation factors.

Similar causal relations to those obtained using DirectLiNGAM analysis are found for results of ICA-LiNGAM. This similarity supports the veracity of the DirectLiNGAM algorithm results. Height appears in the top place in the causal order, as is also the case with DirectLiNGAM. However, the orders of HDL and both of BMI and TG are opposite to those of the DirectLiNGAM. The opposite relation is also apparent between sBP and BMI. To present the causal order clearly, we show Table 5, which presents the causal orders in ICA-LiNGAM estimated from bootstrap samples. Compared with Table 3 in DirectLiNGAM, the BMI variable moved to an earlier order, leading to opposition of the arrows described above.

**Table 5:** Frequencies of orders of various indices in 1000 trials for women in their 70s estimated from ICA-LiNGAM.

Count	1	2	3	4	5	6	7	8	9	10	11
999	height	BMI	sBP	TG	HDL	LDL	GPT	GOT	*γ*GT	fBG	HbA1c
1	height	BMI	sBP	LDL	TG	HDL	GPT	GOT	*γ*GT	fBG	HbA1c

The indices are arranged from earliest to latest in each causal order from left to right columns.

The reason for these differences between the two methods lies in the difference of the estimation method used for the causal order. ICA-LiNGAM determines the causal order using mutual information of the joint distribution of all variables simultaneously, whereas DirectLiNGAM uses score *M* defined in Eq (10), which determines the mutual causal orders successively. The authors of the DirectLiNGAM state that the new method often provides better statistical performance than a state-of-the art method based on ICA [8].

## Comparison with other algorithms

**Fig 9:**
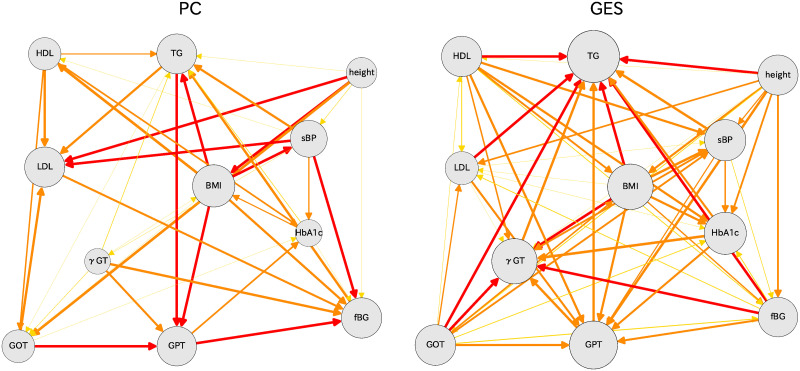
Causal diagrams for women in their 70s: PC (left) and GES (right) algorithms are used for these results.

Other methods are available to assess causal orders of various indices. We chose two methods: PC [17] and GES [18]. The software for these algorithms can be prepared in the ‘pcalg’ package for R [15, 16]. After we performed bootstrap calculations 1000 times, we connected various indices by arrow lines for which thicknesses were obtained using differences of causal directions. The resulting causal relations are shown in Fig 9, where the PC algorithm results are shown as the left hand figure and the GES algorithm results are shown as the right hand figure. Comparison of these two figures reveals many places for which the causal relations differ. Compared to the causal relation with the DirectLiNGAM methods, these two methods show similar skeletons, although some relations are different from those of DirectLiNGAM.

## Conclusion and discussion

The DirectLiNGAM algorithm was applied for analysis of a large amount of health checkup data from Osaka prefecture. As a first trial of this method, 11 indices were used to extract causal relations for men and women of several age groups. Statistically satisfactory results were obtained for the 60s, 70s, and 80s age groups of both men and women, for which the quantities of IDs were more than 30,000 in each group. For samples of 20,000 or smaller, errors in causality become large. The causality relations become fragile.

Based on results of these analyses, several interesting causal relations were found to be quite robust:

- HDL strongly influences BMI and TG; this relation is robust in all age groups.
- LDL is quite independent.
- sBP influences BMI.
- BMI influences fBG (HbA1c) and GPT (*γ*GP).
- TG influences GPT (*γ*GP).
- fBG and HbA1c are correlated strongly, but the causal order is fragile.
- GOP is influenced both by GPT and by *γ*GP.

Several new findings were derived from the LiNGAM analyses presented herein. The role of HDL on BMI and TG is quite important and true for all age groups and for both men and women. However, the role of LDL on other indices is small. These findings must be assessed in greater detail by specifically examining these indices using other statistical methods.

This study represents the first reported application of the DirectLiNGAM to big health checkup data obtained for Osaka prefecture. We used 11 indices for analyses, for which we needed more than 30,000 big data samples. Clear causal relations were obtained among indices. Therefore, it is expected to be very interesting to limit the number of indices and also to try to relate them with illness by selecting medicines to treat certain diseases. As an example, we made calculations of cases with eight variables on the checkup data. We obtained much more robust results than those of the 11-variable cases. We expect to publish more detailed results in future reports. Additionally, we would like to develop a method that includes discrete variables in LiNGAM.

Many possibilities exist for application of DirectLiNGAM analyses of divided data for ‘obese’, ‘normal’, and ‘lean’ groups, respectively representing BMIs of more than 25, 18 through 25, and less than 18. For such cases, the number of indices should be limited strictly in the DirectLiNGAM analysis. We plan to relate the present findings to diseases after several years of checkup data. We expect to approach these interesting problems in studies to be described in future reports.
